# Cerebellar growth, volume and diffusivity in children cooled for neonatal encephalopathy without cerebral palsy

**DOI:** 10.1038/s41598-023-41838-3

**Published:** 2023-09-08

**Authors:** Chelsea Q. Wu, Frances M. Cowan, Sally Jary, Marianne Thoresen, Ela Chakkarapani, Arthur P. C. Spencer

**Affiliations:** 1 Bristol Medical School, University of Bristol, Bristol, UK; 2 Translational Health Sciences, Bristol Medical School, University of Bristol, Bristol, UK; 3 Department of Paediatrics, Imperial College London, London, UK; 4 Faculty of Medicine, Institute of Basic Medical Sciences, University of Oslo, Oslo, Norway; 5 Neonatal Intensive Care Unit, St Michael’s Hospital, University Hospitals Bristol and Weston NHS Foundation Trust, Bristol, BS2 8EG UK

**Keywords:** Paediatric research, Neonatal brain damage

## Abstract

Children cooled for HIE and who did not develop cerebral palsy (CP) still underperform at early school age in motor and cognitive domains and have altered supra-tentorial brain volumes and white matter connectivity. We obtained T1-weighted and diffusion-weighted MRI, motor (MABC-2) and cognitive (WISC-IV) scores from children aged 6–8 years who were cooled for HIE secondary to perinatal asphyxia without CP (cases), and controls matched for age, sex, and socioeconomic status. In 35 case children, we measured cerebellar growth from infancy (age 4–15 days after birth) to childhood. In childhood, cerebellar volumes were measured in 26 cases and 23 controls. Diffusion properties (mean diffusivity, MD and fractional anisotropy, FA) were calculated in 24 cases and 19 controls, in 9 cerebellar regions. Cases with FSIQ ≤ 85 had reduced growth of cerebellar width compared to those with FSIQ > 85 (*p* = 0.0005). Regional cerebellar volumes were smaller in cases compared to controls (*p* < 0.05); these differences were not significant when normalised to total brain volume. There were no case–control differences in MD or FA. Interposed nucleus volume was more strongly associated with IQ in cases than in controls (*p* = 0.0196). Other associations with developmental outcome did not differ between cases and controls.

## Introduction

Neonatal hypoxic-ischaemic encephalopathy (HIE), the neurological sequelae of perinatal asphyxia^1^ is treated with therapeutic hypothermia (TH) where an infant’s core temperature is cooled to 33.5 °C for 72 h commencing within 6 h of birth^2^. Though TH improves outcomes, with 86% of children surviving without CP of any grade^3^, HIE is still a major cause of neonatal death and long-term neurodevelopmental disability. Those without CP have poorer outcomes by early-school age compared to age, sex and socioeconomic status matched control children. These include having lower IQ, needing more support in school, and being at increased risk of motor impairment^4–6^, and having widespread supratentorial alterations, including disrupted structural brain connectivity^7–10^ and smaller subcortical structures^11–13^. This is despite these children having normal or only mildly abnormal neonatal MRIs on visual analysis. However, infratentorial brain development has not been assessed in detail in this cohort, despite the important role played by the cerebellum in both motor^14^ and cognitive function^15^.

Previous studies have found cerebellar injury in neonates cooled for HIE, specifically reporting diffusion abnormalities in the dentate nucleus, cerebellar vermis and cerebellar hemispheres^16,17^ and middle and superior cerebellar peduncles^18^. Signal abnormalities have also been reported in the cerebellar vermis in non-cooled infants^19^ and cooled infants who later died^19,20^. Additionally, volumetric and morphological changes in the cerebellum have been reported. One study found rapid decreases in cerebellar vermis height from the first to second week after birth in cooled infants, who had restricted diffusion on ADC maps or high signal-intensity on DWIs in the pons and cerebellum suggestive of pontine and cerebellar injury, whilst the size of the cerebellum increased in the non-HIE control group^21^. Outside the neonatal period, a study has found altered cerebellar development at 2 years of age, showing that cerebellar volume was lower in children cooled for HIE compared to healthy controls, and that this was associated with poor neurological outcomes^22^. Total cortical volume and the segregated volumes of the lobes of the cerebral cortex were comparable between groups, suggesting limited supratentorial involvement. However, to our knowledge, no studies have specifically assessed the cerebellum in cooled children beyond 2 years of age who were not identified as having neurodevelopmental problems at this age. Therefore, it is unknown whether the early cerebellar alterations persist through childhood.

In this case–control study, we used anatomical MRI and diffusion-weighted imaging (DWI) to quantitatively examine cerebellar volume and diffusion properties at 6–8 years of age in children cooled for HIE secondary to perinatal asphyxia who did not develop CP and in age, sex and socioeconomic status matched control children. We compared regional cerebellar volume and diffusivity between cases and controls and investigated case–control differences in the association between these cerebellar measures and cognitive and motor outcome. In cooled children, we also measured the linear growth of the cerebellum from infancy to early childhood.

## Methods and materials

### Participants

This study investigated participants of the ‘CoolMRI’ study^4^, a study of brain structure and developmental outcomes in early school-age children who received therapeutic hypothermia as a neuroprotective intervention for neonatal HIE, and control children matched for age, sex, and socioeconomic status (measured using the index of multiple deprivation as defined for England by the UK Government; www.gov.uk/government/statistics/english-indices-of-deprivation-2019). Informed and written consent was obtained from the parents of participants and assent obtained from the children. Ethical approval was obtained from the North Bristol Research Ethics Committee and the Health Research Authority (REC ID: 15/SW/0148) and all research was performed in accordance with the Declaration of Helsinki.

Case children were born at or above 35 weeks’ gestation, and received TH as standard clinical care for moderate to severe encephalopathy secondary to perinatal asphyxia^2^. We excluded children with known genetic or metabolic disorders, major intracranial haemorrhage, or congenital brain malformation visible on neonatal MRI, additional medical diagnosis apart from HIE or non English speakers. Cases were sequentially selected from those cooled^3^ between October 2007 and November 2012. Cerebral palsy (CP) was ruled out in participants at 2 years through assessment of motor function and neurological examination and this was confirmed at 6–8 years following a standard clinical neurological examination, including assessment of motor function, tone, and reflexes, following the recommendations of the Surveillance of Cerebral Palsy in Europe^23^.

Controls were matched to the case group for age, sex, and socioeconomic status^4^. Controls were recruited through local schools and newsletters circulated at the University of Bristol. The exclusion criteria include less than 35 weeks gestation at birth, a history of any neurological diagnosis, and non English speakers.

### Assessment of developmental outcome at childhood (6–8 years of age)

Cognitive performance was assessed using the Wechsler Intelligence Scale for Children 4th Edition (WISC-IV)^24^, carried out by a psychologist blinded to case–control status and neuroimaging data. Scores from 10 subtests were used to calculate the following 4 domain scores: verbal comprehension, perceptual reasoning, processing speed and working memory. These were combined to produce a full-scale intelligence quotient (FSIQ). FSIQ of 85 or less is defined as cognitive impairment.

Motor function was assessed using the Movement Assessment Battery for Children, Second Edition (MABC-2)^25^, carried out by two separate assessors who were blinded to case–control status and neuroimaging data. Scores from 8 subtests were used to calculate the following 3 subscales: aiming and catching, balance, and manual dexterity. These were combined to give the MABC-2 total score.

### MRI acquisition

Neonatal MRI scans were acquired using a 1.5 T Siemens Symphony (Erlangen, Germany) between 4 and 15 days after birth, during natural sleep following a feed or with chloral hydrate if required. Volumetric T1-weighted images were acquired using a spin echo sequence with the following parameters: echo time (TE) = 7.7 ms, repetition time (TR) = 400 ms, flip angle = 90°, 0.35 × 0.35 × 5.20 mm voxels. For each subject, two T1-weighted images were acquired with high resolution in the sagittal and transverse plane. Neonatal MRIs were qualitatively assessed by an experienced perinatal neurologist for the presence of cerebellar abnormality, defined as abnormal signal intensity, focal lesions, or abnormal size.

MRI scans at childhood (6–8 years of age) were acquired with a Siemens 3 T Magnetom Skyra (Erlangen, Germany), using the scanning sequence and familiarisation procedure previously described^8^. Volumetric T1-weighted images were acquired using the magnetisation-prepared rapid acquisition gradient echo (MPRAGE) sequence using the following parameters: TE = 2.19 ms, TR = 1500 ms, flip angle = 9°, field of view = 234 × 250 mm, 176 slices, 1 mm isotropic voxels, GeneRalized Autocalibrating Partially Parallel Acquisitions (GRAPPA) acceleration factor 4^26^. Diffusion-weighted images were acquired using a multiband echo-planar imaging sequence, under the following parameters: TE = 70 ms, TR = 3150 ms, flip angle = 90°, field of view = 192 × 192 mm, 60 slices, 2 mm isotropic voxels, phase encoding in the anterior–posterior direction, in-plane acceleration factor = 2, through-plane multi-band factor = 2^27–29^. Two sets of images were acquired with b = 1000 s mm^-2^ in 60 diffusion directions, and 8 interspersed b = 0 images with a blip-up/blip-down sequence, totalling 136 images.

### Measurement of cerebellum growth in cases

To allow assessment of cerebellum growth from the neonatal period to childhood, the vermis height and cerebellar width was measured manually from the sagittal and transverse planes respectively of the T1-weighted images at each time point, by an assessor blinded to developmental outcomes (FC).

### Automated measurement of cerebellum structure

A cerebellum atlas was registered to T1-weighted images in order to measure regional cerebellar volumes. By registration to DWI data, diffusion properties also were measured in each cerebellar region. First, childhood T1-weighted images were visually assessed and rejected if they contained severe movement artefacts. They were then denoised using the Advanced Normalization Tools DenoiseImage tool (http://github.com/ANTsX/ANTs)^30^. DWIs were corrected for eddy current distortions and subject movement using Eddy^31^ and Topup^32^ from FMRIB Software Library (FSL, https://fsl.fmrib.ox.ac.uk)^33^, and resultant image quality was checked using the EddyQC tool^34^. Scans were rejected if the root-mean-square of movement and eddy current metrics was greater than one standard deviation above the mean for the whole cohort.

Fractional anisotropy (FA) and mean diffusivity (MD) images were generated for each subject by fitting a tensor model to the DWI data using the weighted least squares method in FSL’s FDT software. These were then aligned to the T1-weighed image by applying a transformation derived from rigid-body registration of the average b = 0 image to the T1-weighted image, using SPM12.

**Figure 1:**
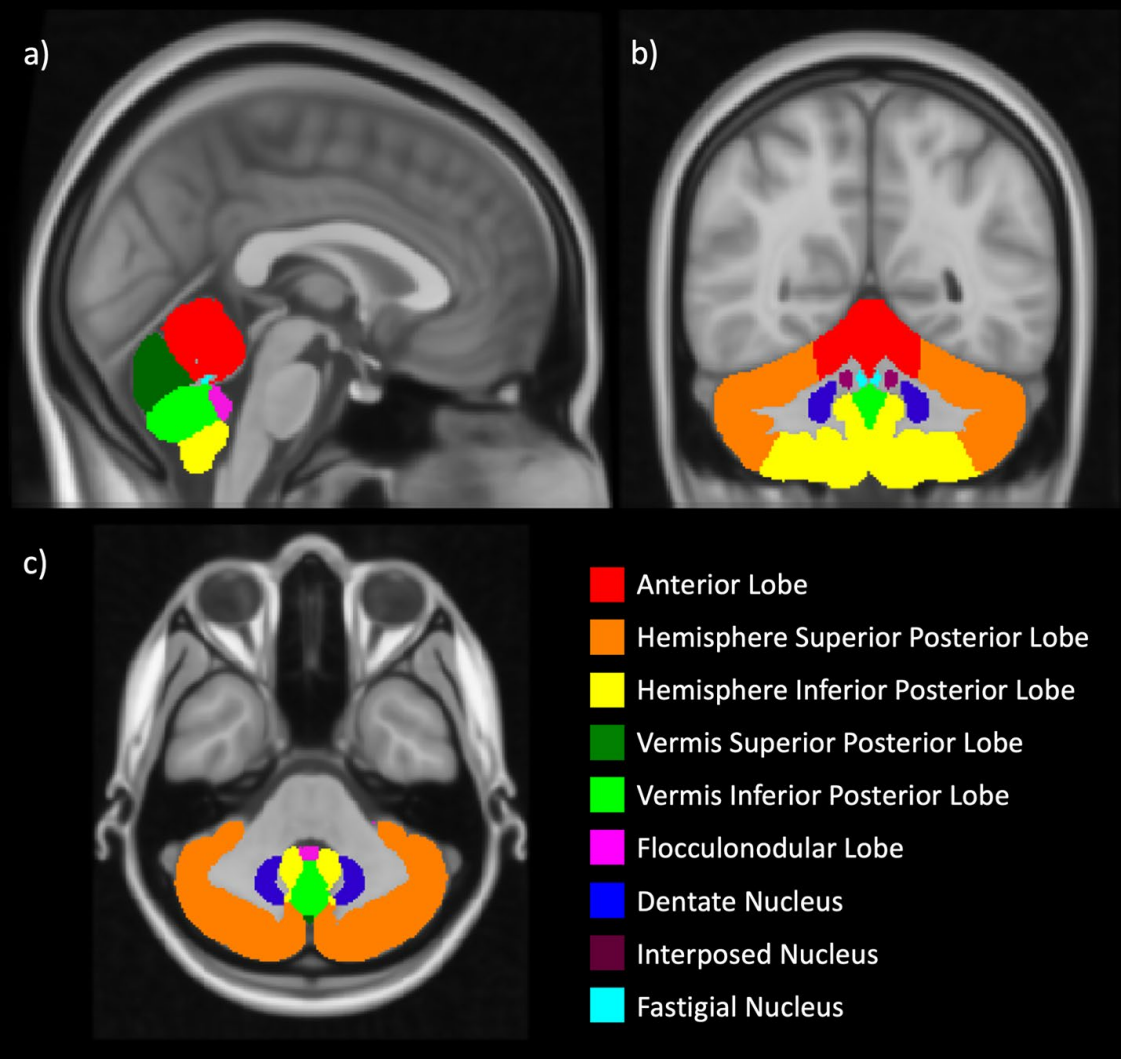
Atlas of the 9 regions of the cerebellum used in our analyses; these are colour coded on the (**a**) sagittal plane, (**b**) coronal, (**c**) axial plane, and overlayed on the MNI standard template.

The cerebellum was automatically segmented to produce an atlas from each subject’s T1-weighted image, using the Spatially Unbiased Atlas Template of the Cerebellum and Brainstem (SUIT) pipeline^35,36^, implemented on SPM12 (https://www.fil.ion.ucl.ac.uk/spm/software/spm12/) on MATLAB (Mathworks, R2021b). The atlas contains 34 cerebellar regions of interest (ROI); given that HIE has a global impact on the brain and does not manifest laterality in brain injury, we combined these into 9 regions, adding together regions in the left and right hemispheres (Fig. 1). Supplementary Table S1 shows how the 34 ROIs from the SUIT atlas were combined to form the 9 regions used in our analysis.

Briefly, the SUIT pipeline is as follows. Firstly, the T1-weighted image was segmented into grey matter and white matter and cropped to isolate the cerebellar structures from the cerebral cortex. These images were then used to create a subject-specific mask of the cerebellum. Following this the subject’s cerebellum was spatially normalised, through non-linear transformation, to the SUIT template^35,36^. The inverse of the transformation matrix produced during normalisation could then be used to bring the SUIT atlas into subject space to form the final subject-specific cerebellar atlas.

The quality of registration of the subject atlases against their respective T1-weighted image was visually checked independently by two researchers (CQW & APCS). In some subjects, alignment was improved by manually editing the cerebellar mask and repeating the registration step. It was agreed by consensus which subjects would be rejected from analysis due to poor alignment of the cerebellar atlas.

The volume of each of the 9 cerebellar regions was calculated for each subject, as well as the volume normalised to total brain volume (TBV), which was calculated as the total volume (including the cerebellum) of whole-brain grey matter and whole-brain white matter. This includes cortical structures, subcortical structures, and the cerebellum. Average FA and average MD was calculated within each region of the cerebellum. During the calculation of average diffusion metrics in each region, voxels which did not contain FA or MD measurements, due to the smaller field of view of DWIs, were ignored. When calculating average diffusion metrics in regions of the cerebellar cortex (specifically the anterior lobe, superior posterior lobe of the hemispheres and vermis, inferior posterior lobe of the hemispheres and vermis, and the flocculonodular lobe), voxels which were not labelled as grey matter by the SUIT segmentation were ignored in order to prevent partial volume effects causing spurious measurements at the boundary between cerebellar grey matter and CSF. After removal of voxels due to DWI field of view and those containing non-grey matter, if fewer than 10% of voxels remained in a region for a given subject the subject was not included in the analysis of diffusion properties for this region.

### Statistical analysis

Age, sex, and socioeconomic status were compared between case and control groups using Mann–Whitney U tests or Fisher’s exact tests.

We assessed whether cases with cognitive impairment had poorer growth of the cerebellum than those without. In the case group, the difference between school-age and neonatal measurements of vermis height and cerebellum width were compared between cases with FSIQ ≤ 85 and cases with FSIQ > 85, using a single-tailed t-test. To account for covariates, we also measured this difference in growth after regressing age and sex at each timepoint.

For each of the automated measures of cerebellar size and diffusivity at childhood (raw volumes, volumes normalised to TBV, FA, and MD), the Kolmogorov–Smirnov test for normality was applied to each cerebellar region. Non-normally distributed metrics were compared between cases and controls using Mann–Whitney U tests. Otherwise, comparisons were made using unpaired two-tailed t-tests. We also compared the total volume of cerebellar lobes and nuclei (the sum of the volumes of all 9 regions shown in Fig. 1), and this total volume normalised to TBV.

Case–control differences in the strength of association of childhood regional cerebellar volumes with childhood FSIQ score and MABC-2 total score were assessed using ANCOVA, with age, sex, and TBV included as covariates. This was repeated to test for case–control differences in the association between FA or MD and FSIQ score or MABC-2 total score, with age and sex included as covariates.

In all tests, false discovery rate (FDR) correction was applied to correct for multiple comparisons across 9 cerebellar regions. FDR-corrected *p* < 0.05 was considered significant.

## Results

### Recruitment

93 participants (50 cases and 43 controls) were recruited for the study. 7 cases and 4 controls did not want to undergo scanning, and 4 cases had incomplete data due to movement during the scan. This gave a total of 78 participants (39 cases and 39 controls) who were scanned. Measurements of neonatal and childhood vermis height was available in 37 cases, and measurements of neonatal and childhood cerebellar width was available in 35 cases.

**Figure 2:**
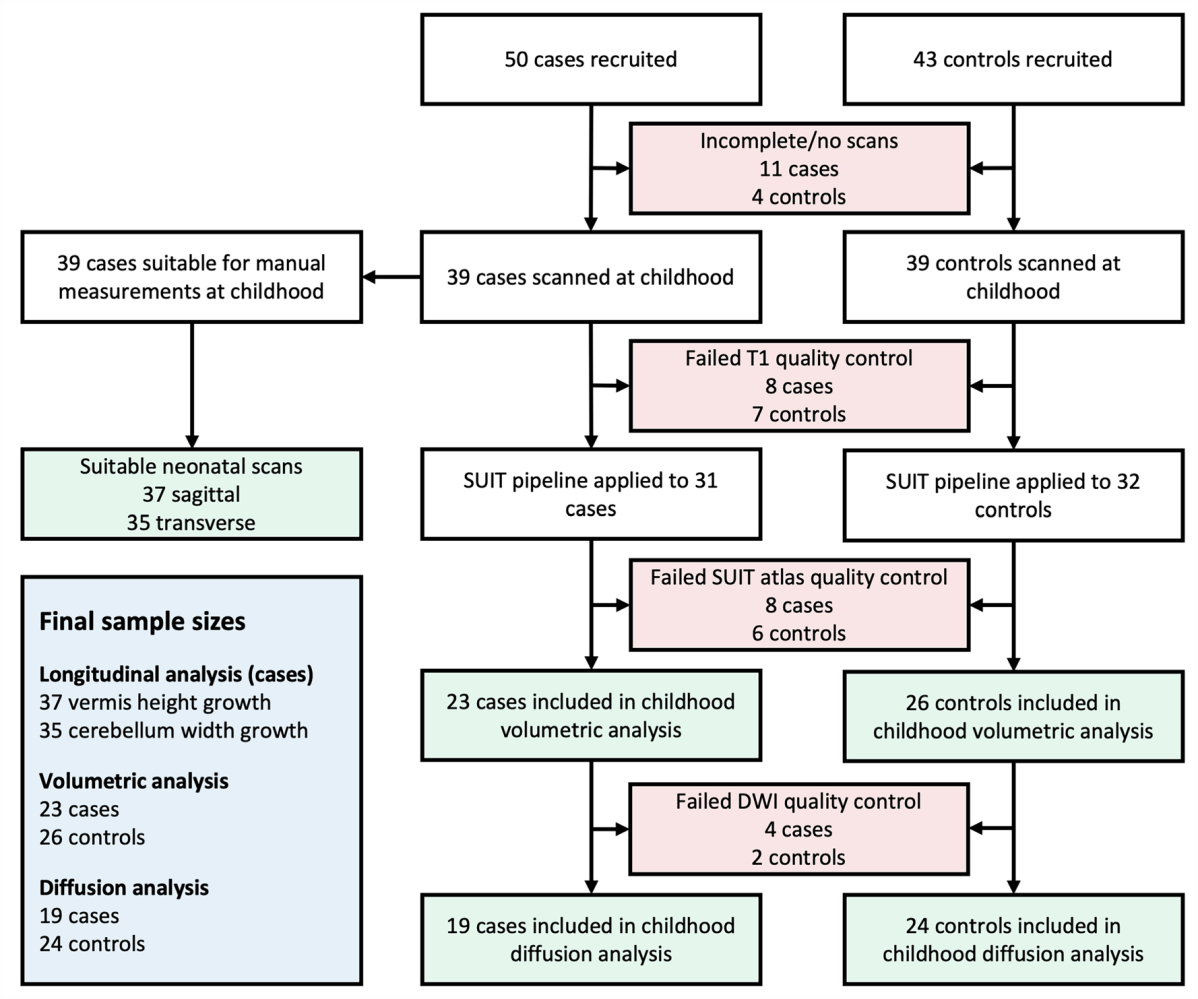
Flowchart of participants at each stage of recruitment and quality control.

Of the 78 subjects scanned at childhood, quality control of T1-weighted images led to the rejection of 8 cases and 7 controls, leaving 63 subjects (31 cases and 32 controls) having T1-weighted images suitable for automated analysis, which were all denoised and processed through the SUIT pipeline. Upon visual inspection of the SUIT segmentation, 5 subjects (3 cases and 2 controls) were improved by manually editing the cerebellar segmentation, while 14 subjects (8 cases, 6 controls) could not be improved and were rejected. This resulted in data of 23 cases and 26 controls being used for volumetric analysis. Of the 49 subjects for whom the cerebellum on the T1-weighted image was segmented and analysed, 1 case did not have any DWI data, and DWI data from 5 subjects (3 cases and 2 controls) did not pass quality control, resulting in 19 cases and 24 controls having DWI data available for statistical analysis. Recruitment and quality control is summarised in Fig. 2.

In one subject, the hemispheres of the inferior posterior lobe only had available diffusion measurements in < 10% of the voxels in the region. This subject was therefore excluded from analysis of this region. In all other regions, all subjects were included.

There were no significant differences in age, sex and socioeconomic status between case and control subjects (Table 1). Only 2 of the 23 cases had cerebellar abnormalities identified through qualitative analysis on neonatal MRI. All other cases had apparently normal cerebellums. There was no significant difference between included or excluded case children in age, socioeconomic status, FSIQ score, MABC-2 total score, or the number of cerebellar abnormalities identified through quantitative analysis of neonatal MRI. However, there was a higher proportion of females in the included case group than the excluded cases (male/female: 8/15 vs 18/9; *p* = 0.0245) (Supplementary Table S2).

**Table 1:** Case–control differences in patient demographics (age, sex, and socioeconomic status measured using the index of multiple deprivation) in the volumetric analysis cohort and diffusion analysis cohort. Total brain volume is also shown for the volumetric analysis cohort.

	Volumetric analysis			Diffusion analysis		
	Case (n = 23)	Control (n = 26)	*p*-value	Case (n = 19)	Control (n = 24)	*p*-value
Age: median (range)	7.2 (6.5–7.9)	7.3 (6.1–7.8)	0.5933	7.0 (6.5–7.9)	7.2 (6.1–7.8)	0.7498
Sex: male/female	8/15	11/15	0.7697	8/11	10/14	1.0
Index of multiple deprivation: median (range)	7 (1–10)	7 (3–10)	0.2936	5 (1–10)	7 (3–10)	0.2622
Total brain volume: mean (standard deviation)/ × 10^3^ cm^3^	1160 (127)	1246 (104)	0.0117	–	–	–

### Cerebellum growth

**Figure 3:**
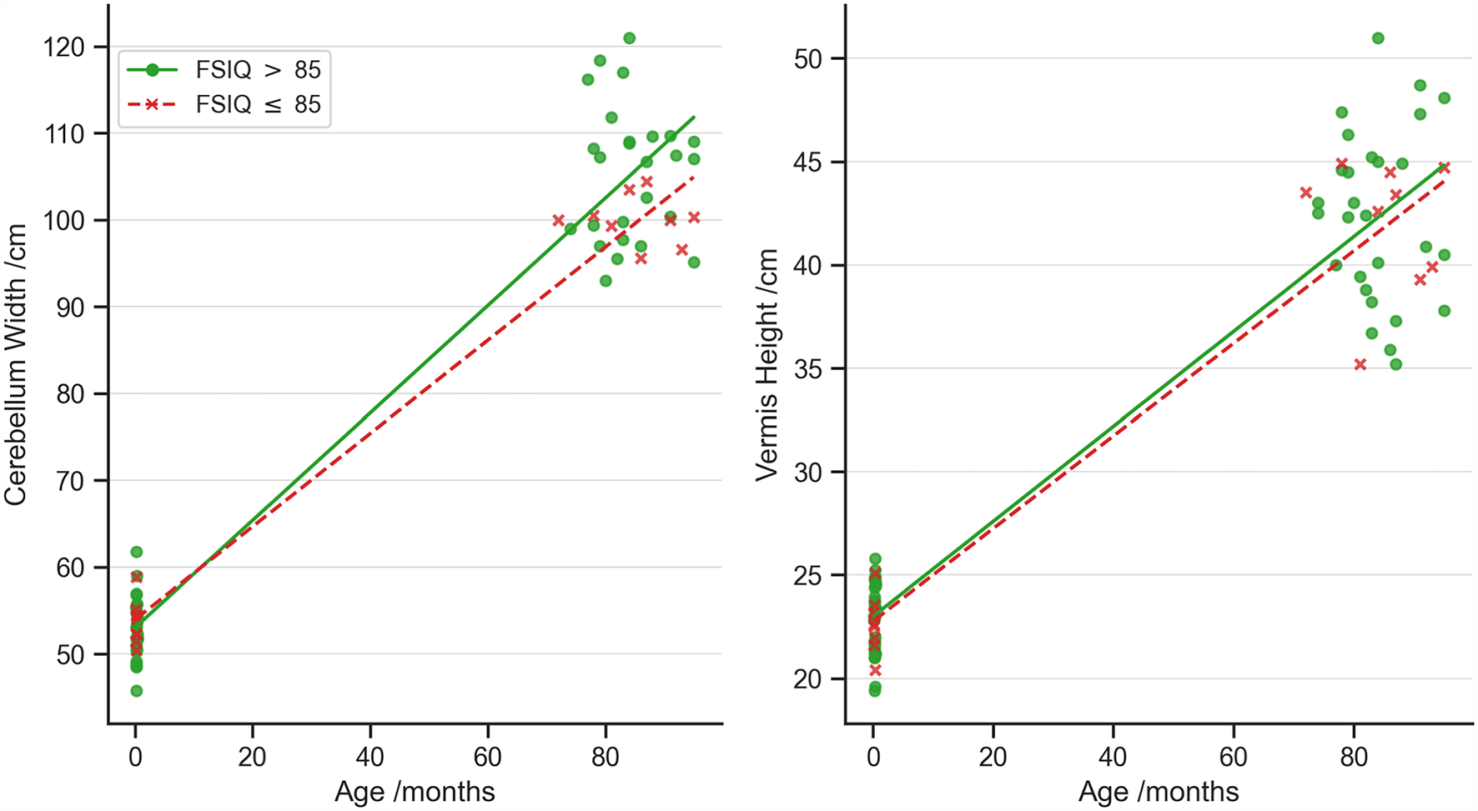
Manual measurements of cerebellar width and vermis height in the neonatal period and at childhood.

The growth of the cerebellum width was significantly lower in cases with FSIQ ≤ 85 compared to cases with FSIQ > 85 (*p* = 0.0005; Fig. 3). This was still significant when age and sex were regressed at each time point prior to calculating growth (*p* = 0.0005). The growth of the vermis height was not significantly different between those with and without cognitive impairment.

### Case–control differences at childhood

**Figure 4:**
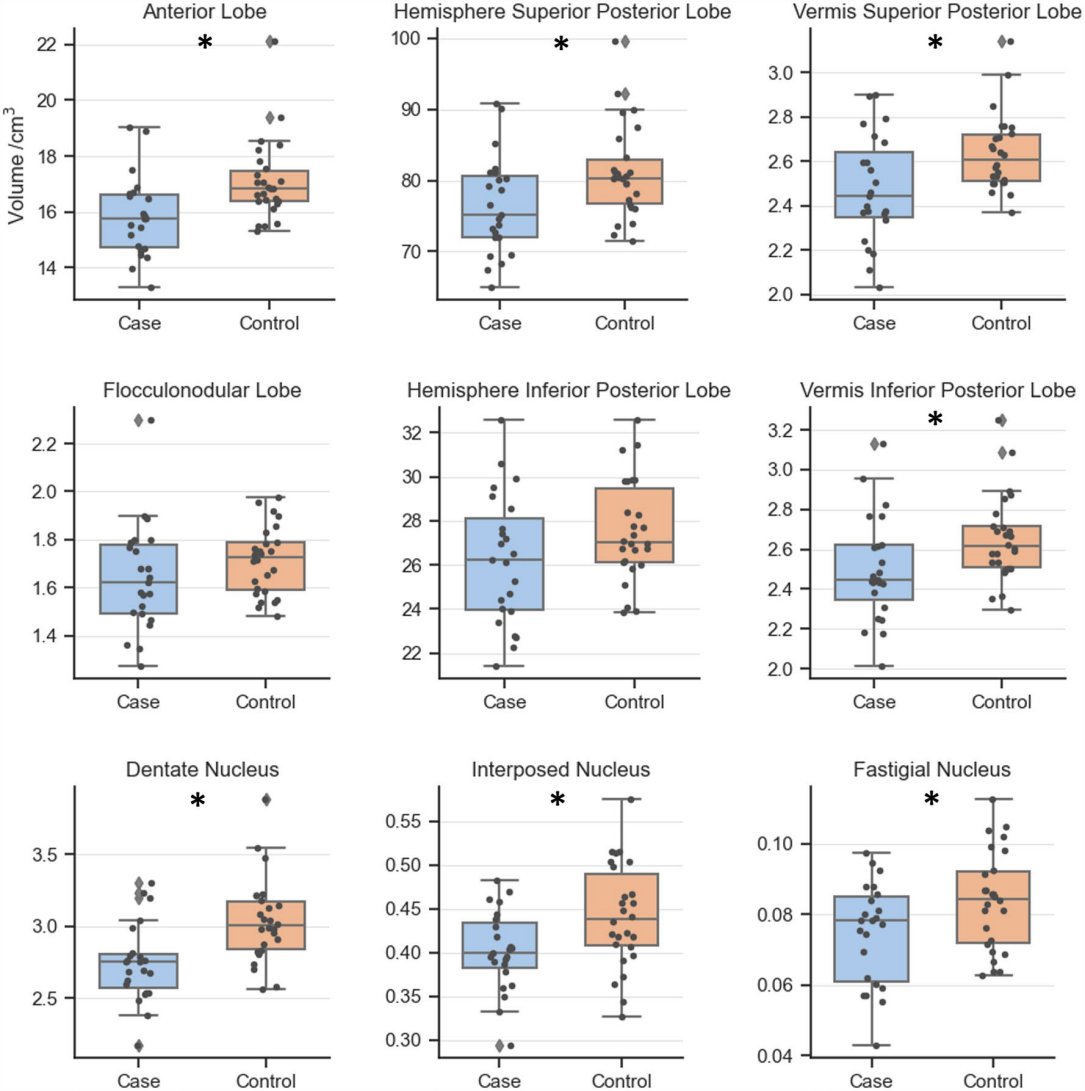
Raw regional cerebellar volumes between case and control groups. The boxes depict the interquartile ranges, with a horizontal line to mark the median value. Whiskers extend to the range of the data, not including outliers shown as diamonds. Overlaid points show the raw data for each subject. *FDR-corrected *p* < 0.05.

Raw cerebellar volumes (i.e. not normalised to TBV) in cases were significantly lower than in controls (Fig. 4) for the following regions: The anterior lobe (15.8 cm^3^ vs 17.1 cm^3^; *p* = 0.0104), hemisphere superior posterior lobe (76.4 cm^3^ vs 81.1 cm^3^; *p* = 0.0378), vermis superior posterior lobe (2.47 cm^3^ vs 2.64 cm^3^; *p* = 0.0255 ), vermis inferior posterior lobe (2.50 cm^3^ vs 2.65 cm^3^; *p* = 0.0440), dentate nucleus (2.74 cm^3^ vs 3.04 cm^3^; *p* = 0.0072), interposed nucleus (0.402 cm^3^ vs 0.442 cm^3^; *p* = 0.0301), and fastigial nucleus (0.0748 cm^3^ vs 0.0838 cm^3^; *p* = 0.0420). Only the hemisphere inferior posterior lobe and flocculonodular lobe were not significantly smaller in cases (see Supplementary Table S3 for group means and *p*-values). There were no case–control differences in manual measurements of cerebellar width or vermis height (*p* > 0.05).

We previously reported reduced whole-brain grey matter and white matter volume in cases compared to controls in this cohort^11^. Table 1 shows that this reduction in TBV is still significant in this subset of the cohort. Therefore, we also investigated regional cerebellar differences after normalisation to TBV, in order to assess whether the differences in cerebellar volumes are independent of the reduction in whole-brain volume. After normalisation to TBV, there were no differences in regional volumes between cases and controls (see Supplementary Table S4 for group means and *p*-values). Total volume of cerebellar lobes and nuclei was lower in cases than in controls (128 cm^3^ vs 136 cm^3^; *p* = 0.0236). However, there was no significant case–control difference in total volume of cerebellar lobes and nuclei after normalisation to TBV (0.111 vs 0.110; *p* = 0.3417).

After FDR-correction, there were no significant case–control differences in FA or MD. See Supplementary Tables S5 and S6 for group means and raw p-values.

### Association with outcomes at childhood

**Figure 5:**
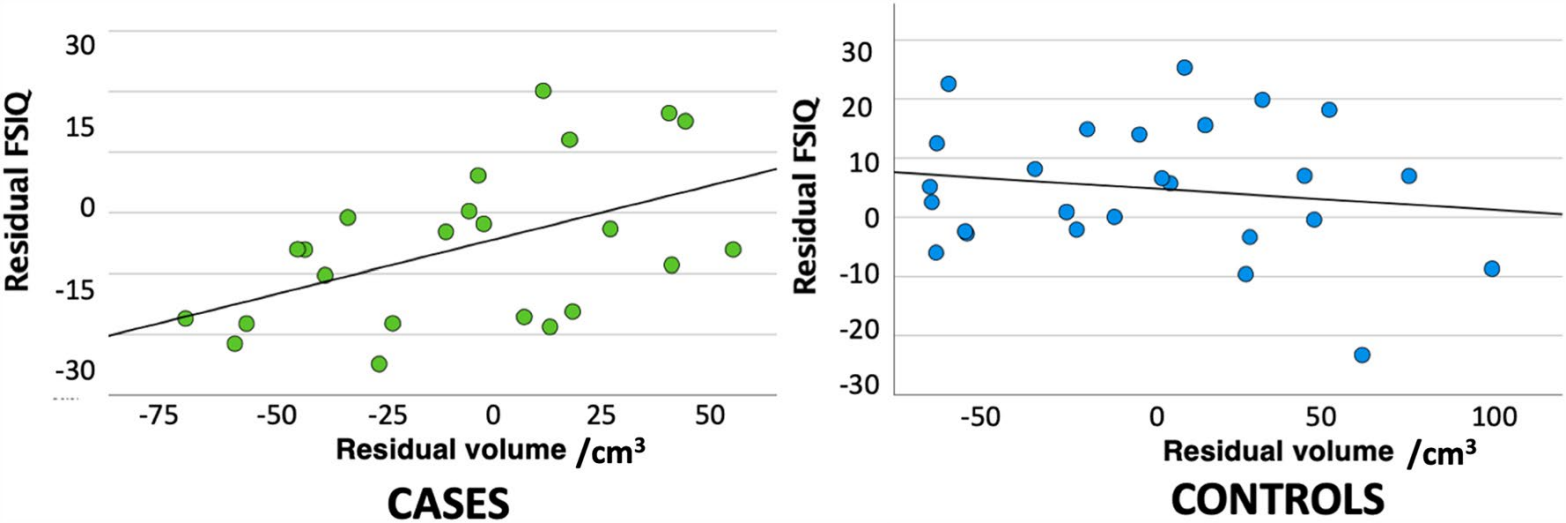
The association between residual interposed nucleus volume and residual FSIQ (composite score derived from WISC-IV subscales) in cases and controls, after controlling for age, sex, and TBV.

The association between raw interposed nucleus volume and FSIQ score was significantly higher in cases than in controls (*p* = 0.0196; Fig. 5), independent of age, sex and TBV. No other case–control differences were found in the association of regional volume with FSIQ score or MABC-2 total score. No case–control differences were found in the association of MD or FA with FSIQ score or MABC-2 total score.

## Discussion

In this study, we investigated the volume, FA and MD in 9 regions of the cerebellum and their associations with motor and cognitive outcomes in early school-aged children without CP who underwent TH for HIE compared to controls matched for age, sex, and socioeconomic status. We found widespread reductions in regional cerebellar volumes in cases compared to controls. There were no case–control differences in regional volumes normalised to TBV, or in FA or MD. FSIQ score was more strongly associated with the volume of the interposed nucleus in cases than in controls, independent of age, sex and TBV. There were no other case–control differences in associations with developmental outcome (FSIQ score or MABC-2 total score). We also measured the growth of the cerebellum from the neonatal period to childhood and found that cooled children with cognitive impairments (defined as FSIQ ≤ 85) had reduced growth of cerebellum width compared to those without.

Previous studies have shown that cerebellar damage from HIE can cause reductions in cerebellar size and growth^21,37^. One study showed a decrease in cerebellar vermis height in cooled infants following cerebellar injury from HIE, compared to an increase in cerebellar vermis height and size in controls from 1 to 2 weeks after birth^21^. Another study showed that neonates with HIE had larger cerebellar sulcal spaces compared to controls at 13–42 days, suggesting poorer development^38^, however this study was conducted prior to the widespread use of TH. Both these studies were conducted on neonates who had cerebellar abnormality seen on visual inspection of the MRI. In our cohort comprising predominantly children with no cerebellar abnormality seen on neonatal MRI, we found widespread reductions in regional cerebellar volume in cases.

There are two possible explanations for the reductions in cerebellar volume in cases. Firstly, development of both supratentorial and cerebellar structures may be disrupted by the same injury mechanism caused by HIE. Hypoxic injury leads to metabolic derangements, inflammation, oxidative stress, glutamate excitotoxicity and neuronal apoptosis and necrosis in brain tissue^39^. This injury occurs at a time that is vital for brain development, and may disrupt plasticity, adaptive potential, brain function, and cause subsequent morphological changes in structure and size of supratentorial and infratentorial structures^40^. Alternatively, it is possible that supratentorial changes have a secondary effect on cerebellar development. In this cohort, FA is reduced in widespread areas of supratentorial white matter^8^, indicating altered white matter connectivity. Neural pathways connecting the cerebral cortex to the cerebellum, including the cerebello-thalamo-cortical (CTC) and cortico-ponto-cerebellar (CPC) pathways, can be found within supratentorial white matter where microstructural damage is evident in children who were cooled for HIE. It is therefore possible that supratentorial damage has had a secondary effect on cerebellar volume and growth.

We found that cooled children with cognitive impairment had reduced growth of the cerebellum compared to those without cognitive impairment, indicating altered developmental trajectory in these children. This mirrors findings of reduced corpus callosum growth across the same timescale in those with cognitive impairment in this cohort^9^. The altered developmental trajectory in cooled children who go on to have poorer cognitive outcomes may be due to alterations resulting from the initial injury, or may arise as a secondary effect due to alterations to other brain areas, as described above. We found no association between cognitive impairment and growth of vermis height in cases, which may be due to difficulty in manually measuring the vermis height.

We found no case–control differences in cerebellar volumes as a proportion of TBV, possibly indicating that the size of each cerebellar region relative to the rest of the brain is comparable between cases and controls, although this interpretation requires caution due to the small sample size and clinically significant differences may have been missed. The reduction in the volume of the whole cerebellum was also not significant when normalized to TBV. The volume of the cerebellum in cases was around 6% lower than in controls; this is comparable to grey matter (7%) and white matter (6%) reductions in cases compared to controls in this cohort, as previously reported^11^. This suggests that volume reductions are widespread throughout the brain and are no more profound in the cerebellum compared to supratentorial regions. The idea that reductions in cerebellar volume likely occur alongside reductions in supratentorial volume is supported by a recent study comparing brain volumes in normotypic children at 2 years of age to those who were cooled for HIE^22^. It was found that total cerebral, temporal lobe, parietal lobe, and cerebellar volumes were reduced in the HIE group. Our recent paper in this cohort also showed widespread decreases in supratentorial volume, including whole-brain grey matter, white matter, pallidi, hippocampi, and thalami volumes in cases compared to controls. Similar to our findings in the cerebellum, subcortical volumes were comparable between cases and controls when normalised to total brain volume^11^. However, unlike the cerebellum, subcortical volumes exhibited associations with developmental outcome in cases but not in controls, independent of age, sex and TBV.

There were no case–control differences in FA or MD, suggesting microstructure in cases is not markedly different to healthy children. Importantly, this is in contrast with supratentorial findings in this cohort that show reductions in FA in widespread areas of white matter, including the fornix, anterior and posterior limbs of the internal capsule, corpus callosum, and cingulum^8,9^. Additionally, we only found case–control differences in the association between the interposed nucleus volume and FSIQ; the associations between developmental outcome and volume, MD, or FA did not differ between cases and controls for all other regions. This absence of substantial case–control differences in cerebellar diffusion properties may suggest cerebellar microstructure is minimally altered in this population, indicating that either the cooling process has offered sufficient protection to preserve cerebellar microstructure, or that any initial microstructural alterations to the cerebellum resulting from hypoxic insult are no longer apparent (or is now so minimal that it was not detected through our analysis) due to adaptations and compensatory mechanisms. However, the lack of significant differences may also be due to the small sample size and FDR correction reducing the power to detect subtle differences. For example, before FDR correction, cases had higher MD than controls in the hemisphere superior posterior lobe and vermis inferior posterior lobe regions (Supplementary Table S5). Though no other regions demonstrated differences with this sample size, future studies with a larger sample size and a hypothesis-driven approach may be sufficiently powered to detect such subtle differences.

Cerebellar microstructural damage is widely reported in infants cooled for HIE, with disrupted diffusion evident through reductions in MD, and FA values^16–18,20^. Abnormal signal intensity in the cerebellum on DWIs have also been identified on qualitative assessment^16^. This damage may result from mechanisms such as cytotoxic oedema, inflammation, and cellular apoptosis, that can cause neuronal damage and restricted diffusion in neonates^41–43^. Additionally, neonatal studies have shown that cerebellar injury from HIE commonly occurs in conjunction with damage to other regions of the brain, including the BGT and brainstem^16,38^. In our case group, in which only 2 of 23 cases had any abnormality seen on neonatal MRI, there could have been underlying microstructural impairments in the other cases which would not be visible upon qualitative assessment, especially as it becomes difficult to see diffusion-weighted changes by the second week of life. Since diffusion properties in cases were comparable to controls at early-school age, if any microstructural damage had been present it has seemingly recovered. This could be due to early adaptations within the cerebellum such as neuronal regeneration and reorganisation due to the neuroplastic capabilities of the developing brain^44^, that have compensated for any damage caused by the initial insult^45^. Thus, in cooled children with non-severe outcomes, it seems that brain development is sufficient to recover from the initial hypoxic insult to the cerebellum.

The volume of the interposed nucleus was more strongly associated with FSIQ in cases than in controls. The interposed nucleus is thought to be involved in conditioning of discrete, somatic muscle responses such as eyelid responses and gait kinematics^46^. Sensory functions have also been described, with one study showing the interposed nucleus responds to tactile stimulation^47^. Whilst there is no literature specifically linking the interposed nucleus to cognition, the cerebellum is highly involved in cognitive function and the deep cerebellar nuclei, including the interposed nucleus, form the output from the cerebellum to other regions of the brain such as the limbic system and cerebral cortex.

### Limitations

The main limitation of this study is the small sample size. Due to the inherent difficulty involved with scanning children of this age group, many scans were not of sufficient quality to allow investigation of the cerebellum at childhood, resulting in a high percentage of enrolled children who were not included in the analysis. We have previously shown that motion artefacts on brain MRI in this cohort are related to developmental profile^48^ thus there may be some selection bias associated with the rejection of those with poor quality MRI scans. However, the included case group was comparable to rejected cases in terms of age, socioeconomic status, FSIQ score, MABC-2 total score and the rate of neonatal cerebellar abnormalities. Thus, our case group is representative of the wider population of cooled children we recruited, in terms of demographics (apart from sex) and outcomes.

In the longitudinal investigation of cerebellar growth, our analysis was limited to measurements of cerebellar width and vermis height due to the thick-slice anatomical scans acquired in the neonatal period. In future studies of cooled infants, higher resolution anatomical scans, in addition to DWI data, could be acquired to facilitate analysis of cerebellar volumes and microstructure in neonates. This may determine whether the altered development of the cerebellum is due to initial alterations resulting from the injury, or is a secondary effect of supratentorial alterations. In turn, this may indicate whether further neuroprotection of the cerebellum is required at birth, or whether support may be offered during development to promote healthy cerebellar growth.

For automated parcellation of the cerebellum at childhood, we used the SUIT atlas as there is currently no publicly available paediatric cerebellar atlas. The SUIT atlas was created from scans of 20 adults with a mean age of 27.25 years^35,36^. Using an adult atlas gives a less accurate representation of anatomical features than an age-specific atlas^49^. For example, SUIT overestimates lobule VI, found in the right, left, and vermis of the superior posterior lobe in this study, which is proportionally smaller in children compared to adults, whilst SUIT often underestimates lobule IX, found in the right, left and vermis of the inferior posterior lobe in this study, which is proportionally larger in children^50^.

Finally, we took an exploratory approach when investigating volume and diffusivity in cerebellar regions at childhood; we measured volume and diffusion properties in 9 cerebellar regions and corrected for multiple comparisons, as the cerebellum has not been sufficiently explored in cooled children to focus our analyses on specific regions. As discussed above, future studies may take a hypothesis-driven approach in order to increase statistical power.

## Conclusion

To summarise, our results show that children who had been cooled for HIE with cognitive impairment (FSIQ ≤ 85) but without CP at early school age have poorer cerebellar development than those without cognitive impairment (FSIQ > 85). Additionally, these children have smaller cerebellar volumes than healthy controls. However, our results suggest TH provides sufficient protection of the cerebellum to allow healthy microstructural development such that, at early school-age, there are minimal differences in the diffusion properties of the cerebellum between cooled children without CP and healthy controls.

### Supplementary Information

Supplementary Table S1.

Supplementary Table S2.

Supplementary Table S3.

Supplementary Table S4.

Supplementary Table S5.

Supplementary Table S6.

## Data Availability

The datasets generated during and/or analysed during the current study are available from the corresponding author on reasonable request.
