# Author Correction: Cerebellar growth, volume and diffusivity in children cooled for neonatal encephalopathy without cerebral palsy

**DOI:** 10.1038/s41598-023-49211-0

**Published:** 2023-12-29

**Authors:** Chelsea Q. Wu, Frances M. Cowan, Sally Jary, Marianne Thoresen, Ela Chakkarapani, Arthur P. C. Spencer

**Affiliations:** 1 Bristol Medical School, University of Bristol, Bristol, UK; 2 Translational Health Sciences, Bristol Medical School, University of Bristol, Bristol, UK; 3 Department of Paediatrics, Imperial College London, London, UK; 4 Faculty of Medicine, Institute of Basic Medical Sciences, University of Oslo, Oslo, Norway; 5 Neonatal Intensive Care Unit, St Michael’s Hospital, University Hospitals Bristol and Weston NHS Foundation Trust, Bristol, BS2 8EG UK

Correction to: *Scientific Reports* 10.1038/s41598-023-41838-3, published online 08 September 2023

The original version of this Article contained an error in Figure 1, where the colour labels of 'Fastigial Nucleus' and 'Interposed Nucleus' were interchanged. The original Figure 1 and accompanying legend appear below.

**Figure 1:**
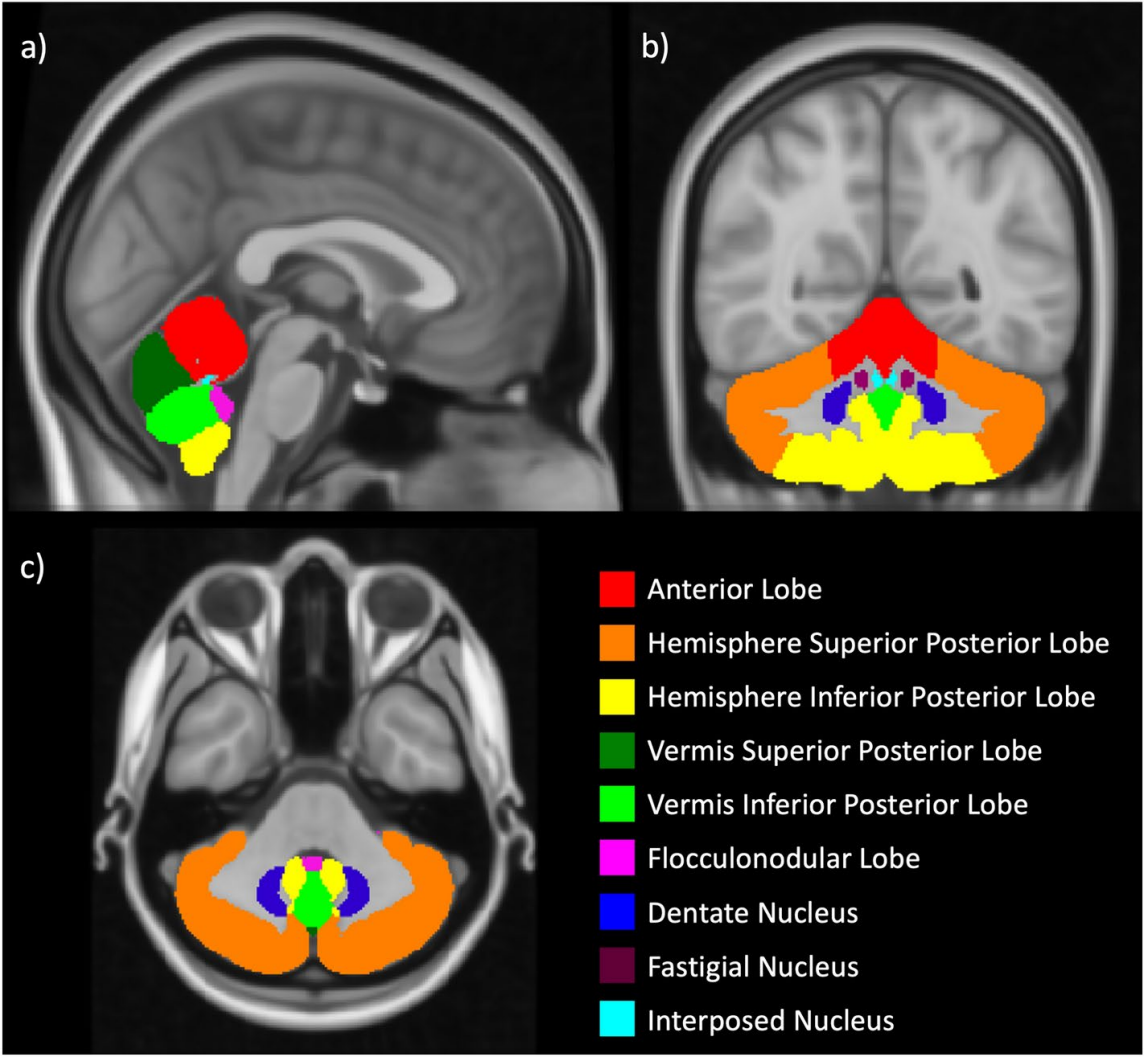
Atlas of the 9 regions of the cerebellum used in our analyses; these are colour coded on the (**a**) sagittal plane, (**b**) coronal, (**c**) axial plane, and overlayed on the MNI standard template.

The original Article has been corrected.

