# Supplementary material for: Cerebellar growth, volume and diffusivity in children cooled for neonatal encephalopathy without cerebral palsy
Source: Sci Rep. 2023 Sep 8;13:14869. doi: 10.1038/s41598-023-41838-3 (PMC10491605; doi:10.1038/s41598-023-41838-3)
Supplement: Supplementary file 1 — Supplementary Table S1. [file 41598_2023_41838_MOESM1_ESM.docx]

Supplementary Table S1: This table shows how each of the 34 cerebellar regions labelled by SUIT were combined to form the 9 larger regions used in our analysis.

| **Cerebellar Region** | **SUIT regions** |
| --- | --- |
| Anterior Lobe | I_IV (R & L)  V (R & L) |
| Hemisphere Superior Posterior Lobe | VI (R & L)  Crus I (R & L)  Right Crus II (R & L)  Right VIIb (R & L) |
| Hemisphere Inferior Posterior Lobe | VIIIa (R & L)  VIIIb (R & L)  IX (R & L) |
| Vermis Superior Posterior Lobe | Vermis VI  Vermis Crus I  Vermis Crus II  Vermis VIIb |
| Vermis Inferior Posterior Lobe | Vermis VIIIa  Vermis VIIIb  Vermis IX |
| Flocculonodular Lobe | X (R & L)  Vermis X |
| Dentate nucleus | Dentate nucleus (R & L) |
| Interposed nucleus | Interposed nucleus (R & L) |
| Fastigial nucleus | Fastigial nucleus (R & L) |
