# Supplementary material for: Cerebellar growth, volume and diffusivity in children cooled for neonatal encephalopathy without cerebral palsy
Source: Sci Rep. 2023 Sep 8;13:14869. doi: 10.1038/s41598-023-41838-3 (PMC10491605; doi:10.1038/s41598-023-41838-3)
Supplement: Supplementary file 2 — Supplementary Table S2. [file 41598_2023_41838_MOESM2_ESM.docx]

Supplementary Table S2: Comparison of demographics between cases included in the volumetric analysis and those rejected. n.s. = not significant.

|  | **Accepted cases**  **(n=25)** | **Rejected cases**  **(n=27)** | **p-value** |
| --- | --- | --- | --- |
| **Age: median (range)** | 7.2 (6.5-7.9) | 6.9 (6.0-7.9) | n.s. |
| **Sex: male/female** | 8/15 | 18/9 | 0.0245 |
| **Index of multiple deprivation: median (range)** | 7 (1-10) | 6 (2-10) | n.s. |
| **FSIQ score: median (range)** | 92 (62-123) | 98 (81-114) | n.s. |
| **MABC total score: median (range)** | 10 (3-19) | 9 (1-15) | n.s. |
| **Neonatal cerebellar abnormalities: yes/no** | 2/21 | 1/26 | n.s. |
