# Supplementary material for: Cerebellar growth, volume and diffusivity in children cooled for neonatal encephalopathy without cerebral palsy
Source: Sci Rep. 2023 Sep 8;13:14869. doi: 10.1038/s41598-023-41838-3 (PMC10491605; doi:10.1038/s41598-023-41838-3)
Supplement: Supplementary file 3 — Supplementary Table S3. [file 41598_2023_41838_MOESM3_ESM.docx]

Supplementary Table S3: Mean volumes of cerebellar regions in cases compared to controls. Also shown are raw p-values from case-control comparison, and FDR-corrected p-values. n.s. = not significant.

| **Cerebellar region** | **Case mean** | **Control mean** | **P-value** | **Corrected P-value** |
| --- | --- | --- | --- | --- |
| Anterior lobe | 15833 | 17091 | 0.0025 | 0.0112 |
| Hemisphere superior posterior lobe | 76438 | 81124 | 0.0210 | 0.0382 |
| Hemisphere inferior posterior lobe | 26228 | 27556 | n.s. | n.s. |
| Vermis superior posterior lobe | 2474.7 | 2637.8 | 0.0085 | 0.0256 |
| Vermis inferior posterior lobe | 2501.8 | 2651.3 | 0.0342 | 0.0440 |
| Flocculonodular lobe | 1640.6 | 1712.3 | n.s. | n.s. |
| Dentate nucleus | 2744.6 | 3036.4 | 0.0008 | 0.0068 |
| Interposed nucleus | 402.37 | 441.74 | 0.0134 | 0.0302 |
| Fastigial nucleus | 74.635 | 83.842 | 0.0280 | 0.0420 |
