# Supplementary material for: Cerebellar growth, volume and diffusivity in children cooled for neonatal encephalopathy without cerebral palsy
Source: Sci Rep. 2023 Sep 8;13:14869. doi: 10.1038/s41598-023-41838-3 (PMC10491605; doi:10.1038/s41598-023-41838-3)
Supplement: Supplementary file 4 — Supplementary Table S4. [file 41598_2023_41838_MOESM4_ESM.docx]

Supplementary Table S4: Mean normalised volumes (raw volumes divided by TBV) of cerebellar regions in cases and controls. No regions were significant before FDR correction so corrected p-values are not shown.

| **Cerebellar region** | **Case mean** | **Control mean** | **P-value** |
| --- | --- | --- | --- |
| Anterior lobe | 13.71 x 10^-3^ | 13.73 x 10^-3^ | n.s. |
| Hemisphere superior posterior lobe | 66.17 x 10^-3^ | 65.14 x 10^-3^ | n.s. |
| Hemisphere inferior posterior lobe | 22.65 x 10^-3^ | 22.14 x 10^-3^ | n.s. |
| Vermis superior posterior lobe | 2.143 x 10^-3^ | 2.121 x 10^-3^ | n.s. |
| Vermis inferior posterior lobe | 2.165 x 10^-3^ | 2.131 x 10^-3^ | n.s. |
| Flocculonodular lobe | 1.416 x 10^-3^ | 1.377 x 10^-3^ | n.s. |
| Dentate nucleus | 2.374 x 10^-3^ | 2.441 x 10^-3^ | n.s. |
| Interposed nucleus | 0.348 x 10^-3^ | 0.355 x 10^-3^ | n.s. |
| Fastigial nucleus | 64.2 x 10^-6^ | 67.0 x 10^-6^ | n.s. |
