# Supplementary material for: Cerebellar growth, volume and diffusivity in children cooled for neonatal encephalopathy without cerebral palsy
Source: Sci Rep. 2023 Sep 8;13:14869. doi: 10.1038/s41598-023-41838-3 (PMC10491605; doi:10.1038/s41598-023-41838-3)
Supplement: Supplementary file 5 — Supplementary Table S5. [file 41598_2023_41838_MOESM5_ESM.docx]

Supplementary Table S5: The mean MD of each region of the cerebellum, compared between cases and controls. Also shown are uncorrected p-values from case-control comparison and FDR-corrected p-values. One subject was excluded from analysis of the hemispheres of the inferior posterior lobe as <10% of voxels had diffusion measurements available. n.s. = not significant.

| **Cerebellar region** | **Case mean MD** | **Control mean MD** | **P-value** | **Corrected P-value** |
| --- | --- | --- | --- | --- |
| Anterior Lobe | 0.825 x 10^-3^ | 0.805 x 10^-3^ | n.s | n.s. |
| Hemisphere Superior Posterior Lobe | 0.745 x 10^-3^ | 0.726 x 10^-3^ | 0.0282 | n.s. |
| Hemisphere Inferior posterior lobe | 0.712 x 10^-3^ | 0.680 x 10^-3^ | n.s. | n.s. |
| Vermis superior posterior lobe | 0.819 x 10^-3^ | 0.802 x 10^-3^ | n.s. | n.s. |
| Vermis inferior posterior lobe | 0.812 x 10^-3^ | 0.790 x 10^-3^ | 0.0233 | n.s. |
| Flocculonodular lobe | 0.993 x 10^-3^ | 0.932 x 10^-3^ | n.s. | n.s. |
| Dentate nucleus | 0.781 x 10^-3^ | 0.785 x 10^-3^ | n.s. | n.s. |
| Interposed nucleus | 0.827 x 10^-3^ | 0.837 x 10^-3^ | n.s. | n.s. |
| Fastigial nucleus | 0.841 x 10^-3^ | 0.846 x 10^-3^ | n.s. | n.s. |
