# Supplementary material for: Cerebellar growth, volume and diffusivity in children cooled for neonatal encephalopathy without cerebral palsy
Source: Sci Rep. 2023 Sep 8;13:14869. doi: 10.1038/s41598-023-41838-3 (PMC10491605; doi:10.1038/s41598-023-41838-3)
Supplement: Supplementary file 6 — Supplementary Table S6. [file 41598_2023_41838_MOESM6_ESM.docx]

Supplementary Table S6: The mean FA of each region of the cerebellum, compared between cases and controls. Also shown are uncorrected p-values from case-control comparison. No regions were significant before FDR correction so corrected p-values are not shown. One subject was excluded from analysis of the hemispheres of the inferior posterior lobe as <10% of voxels had diffusion measurements available. n.s. = not significant.

| **Cerebellar region** | **Case mean FA** | **Control mean FA** | **P-value** |
| --- | --- | --- | --- |
| Anterior Lobe | 0.196 | 0.201 | n.s. |
| Superior Posterior Lobe | 0.200 | 0.205 | n.s. |
| Inferior posterior lobe | 0.188 | 0.197 | n.s. |
| Vermis superior posterior lobe | 0.172 | 0.167 | n.s. |
| Vermis inferior posterior lobe | 0.178 | 0.187 | n.s. |
| Flocculonodular lobe | 0.136 | 0.160 | n.s. |
| Dentate nucleus | 0.298 | 0.305 | n.s. |
| Interposed nucleus | 0.307 | 0.320 | n.s. |
| Fastigial nucleus | 0.233 | 0.223 | n.s. |
